# Supplementary material for: Protective Effects of Fucoidan on Iodoacetamide-Induced Functional Dyspepsia via Modulation of 5-HT Metabolism and Microbiota
Source: Int J Mol Sci. 2025 Apr 1;26(7):3273. doi: 10.3390/ijms26073273 (PMC11989908; doi:10.3390/ijms26073273)
Supplement: Supplementary file 1 [file ijms-26-03273-s001.zip › ijms-3489753-supplementary.pdf]

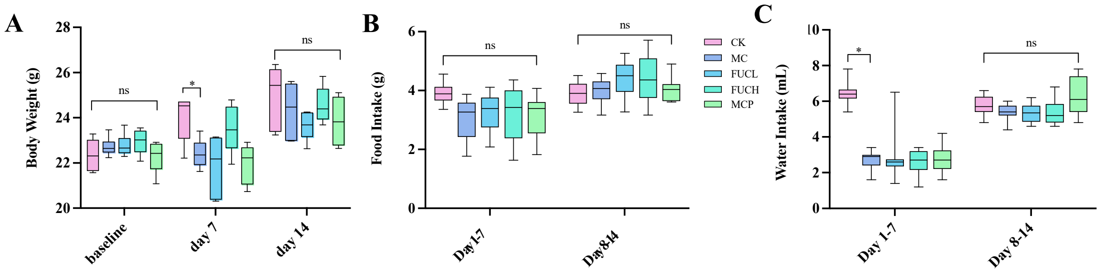

Figure S1. The general physiological state of mice in each group. During the 14 days, the body weight (A), food intake (B), and water intake (C) were observed and recorded.
